# Abnormal Circadian Modification of A*δ*-Fiber Pathway Excitability in Idiopathic Restless Legs Syndrome

**DOI:** 10.1155/2019/5408732

**Published:** 2019-11-03

**Authors:** Catello Vollono, Giacomo Della Marca, Elisa Testani, Anna Losurdo, Daniela Virdis, Diana Ferraro, Valerio Brunetti, Paolo M. Rossini, Domenica Le Pera, Salvatore Mazza, Massimiliano Valeriani

**Affiliations:** ^1^Unit of Neurophysiopathology, Department of Geriatrics, Neurosciences and Orthopedics, Catholic University, Policlinico Universitario “A. Gemelli” IRCCS, Rome, Italy; ^2^Neurology Unit, Department of Neurosciences, University of Modena and Reggio Emilia, Modena, Italy; ^3^Area Neuroscienze, San Raffaele Pisana IRCCS, Rome, Italy; ^4^Neurology Division, Pediatric Hospital “Bambino Gesù” IRCCS, Rome, Italy; ^5^Center for Sensory-Motor Interaction, Aalborg University, Aalborg, Denmark

## Abstract

Restless legs syndrome (RLS) is characterized by unpleasant sensations generally localized to legs, associated with an urge to move. A likely pathogenetic mechanism is a central dopaminergic dysfunction. The exact role of pain system is unclear. The purpose of the study was to investigate the nociceptive pathways in idiopathic RLS patients. We enrolled 11 patients (mean age 53.2 ± 19.7 years; 7 men) suffering from severe, primary RLS. We recorded scalp laser-evoked potentials (LEPs) to stimulation of different sites (hands and feet) and during two different time conditions (daytime and nighttime). Finally, we compared the results with a matched control group of healthy subjects. The A*δ* responses obtained from patients did not differ from those recorded from control subjects. However, the N1 and the N2-P2 amplitudes' night/day ratios after foot stimulation were increased in patients, as compared to controls (N1: patients: 133.91 ± 50.42%; controls: 83.74 ± 34.45%; *p* = 0.016; A*δ*-N2-P2: patients: 119.15 ± 15.56%; controls: 88.42 ± 23.41%; *p* = 0.003). These results suggest that RLS patients present circadian modifications in the pain system, which are not present in healthy controls. Both sensory-discriminative and affective-emotional components of pain experience show parallel changes. This study confirms the structural integrity of A*δ* nociceptive system in idiopathic RLS, but it also suggests that RLS patients present circadian modifications in the pain system. These findings could potentially help clinicians and contribute to identify new therapeutic approaches.

## 1. Introduction

Idiopathic restless legs syndrome (RLS) is a large prevalent chronic sensory-motor disorder [1] and it is characterized by unpleasant sensations generally localized to legs and associated with an urge to move. The etiology of RLS is not fully understood. The most likely pathogenic mechanism consists in a central dopaminergic dysfunction. The symptoms worsen or are exclusively present at rest [1]. RLS symptoms have a circadian rhythm: they reach maximal severity at night, when the dopamine levels are at their nadir, with consequent disruption of sleep [2]. The motor symptoms, represented by an incoercible impulse to move the legs and repetitive, spontaneous, stereotyped, voluntary, and unintentional movements of the legs, are present during both wake and sleep [3].

Although there is no agreement about the prevalence of pain in RLS, some RLS patients describe their sensations as painful [4]. Involvement of nociception in RLS is also suggested by the therapeutic response to opioids and the physiologic link between dopamine and pain control [5].

The exact role of pain system in RLS is unclear and only few studies addressed this issue. In patients with RLS, both primary and secondary to large fiber neuropathy, Schattschneider et al. found an impairment of thermal perception threshold suggesting a small fiber involvement [6]. Another study [7] demonstrated that symptomatic RLS may be triggered by small fiber neuropathy. However, no epidermal fiber abnormality was found in skin biopsies of patients with idiopathic RLS [6]. The lower pain threshold in RLS patients than in control suggests that pain processing may be amplified in this disease [8]. Lastly, hyperalgesia associated with tactile hypoesthesia and paradoxical heat sensation was described in RLS patients [9].

Laser-evoked potential (LEP) recording represents the most reliable neurophysiological technique to assess the human nociceptive system function (evidence level A) [10]. CO_2_ laser pulses delivered on the skin activate the nociceptive A*δ* and C fibers, without any stimulation of the nonnociceptive A*β* afferents [11].

To the best of our knowledge, only one previous study assessed the nociceptive function in RLS by using LEPs [12]. No abnormalities of both LEPs and sympathetic skin responses were found. However, the neurophysiological responses were recorded in one session; thus, the characteristic circadian rhythm of the symptoms in RLS was not considered.

The aims of the present study were to investigate (1) the function of the A∂-fiber pathway in idiopathic RLS patients by recording LEPs to stimulation of different sites (hands and feet) and (2) possible modifications of the nociceptive system excitability linked to the circadian rhythm of the disease. To reach this purpose, LEPs were recorded at two different times (early afternoon and nighttime).

## 2. Methods

### 2.1. Patients and Healthy Controls

We enrolled 11 patients (mean age 53.2 ± 19.7 years; 7 men, 4 women, range 22–77 years; disease duration: 13.2 ± 15.6 months). RLS diagnosis was reached by using a structured interview following the International Restless Legs Syndrome Study Group (IRLSSG) diagnostic criteria [13]. Severity of RLS symptoms was measured by the IRLSSG scale [13].

Inclusion criteria were (1) primary RLS, (2) severe symptoms (IRLSSG score > 15, mean = 30.0 ± 3.8), (3) exclusive or prevalent involvement of lower limbs, and (4) presence of a definite circadian pattern of symptoms. Exclusion criteria were (1) other movement disorders, (2) other sleep disorders, (3) neurological or medical disease, (4) any condition associated with chronic musculoskeletal or neuropathic pain, (5) intake of dopamine agonists, antidopaminergic, as well as other neurological active drugs (benzodiazepines, opioids, GABA-agonists, etc.), (6) any condition possibly causing secondary RLS (renal disease, anemia, and pregnancy), and (7) history of alcohol or drug abuse. All patients underwent full medical and neurological examination and neurophysiological tests, including measurements of nerve conduction, somatosensory-evoked potentials, motor-evoked potentials during daytime and overnight, and laboratory-based polysomnography. The main clinical data concerning the patients are summarized in Table 1. Clinical and instrumental data obtained from patients were compared with those obtained from 11 healthy volunteers, matched for sex and age (mean age 55.4 ± 18.7 years; 7 males, 4 females, range 21–74 years). Data concerning the control subjects are reported in Table 2. The study was approved by the local ethical committee, and all subjects gave their informed consent to participate.

### 2.2. Laser Stimulation and LEP Recording

In both patients and controls, LEP recordings were performed in two separate sessions: daytime session (on early afternoon, between 1:00 PM and 3:00 PM) and nighttime session (on evening, between 9:00 PM and 11:00 PM). LEPs were obtained to stimulation of both hand and foot. Right and left sides were stimulated. Two averages of 20 trials each were obtained for each session and each stimulation site.

During LEP recording, the subject lay on a couch in a warm and semidarkened room. Cutaneous heat stimuli were delivered by a CO_2_ laser (wavelength 10.6 *μ*m, beam diameter 2 mm, duration 10 milliseconds, CO_2_ Neurolas Electronic Engineering, Florence, Italy). The stimulation site was visualized by a He-Ne laser beam.

The location of the impact on the skin was slightly shifted between two successive stimuli to avoid the sensitization of the nociceptors. The sensory threshold (STh), defined as the lower stimulus intensity eliciting a distinct pinprick sensation, was determined by the method of limits in three series of increasing and decreasing stimulus intensities. To record LEPs, we used an intensity set at 2.5x STh (recording intensity, RI), which was felt as a painful pinprick by all the subjects. The interstimulus interval (ISI) varied randomly between 8 and 12 seconds. All subjects underwent a standard recording session simultaneously gathered from 3 scalp electrodes placed on Fz, Cz, and contralateral temporal region (T3 or T4), defined according to the 10–20 International System of EEG recording. The reference electrode was placed at the nose and the ground on the forehead (Fpz). Eye movements and eye blinks were monitored by electro-oculogram (EOG). Signals were amplified, filtered (bandpass 0.3–70 Hz), and stored for offline average and analysis. The analysis time was 1000 milliseconds with a bin width of 2 milliseconds. An automatic artifact rejection system excluded from the average procedure all trials contaminated by transients exceeding ±65 *μ*V at any recording channel, including EOG. In order to ensure that the attention level of subjects did not change across the whole experiment, they were asked to count the number of the received laser stimuli silently. Any recording with a counting mistake higher than 10% would not be considered for further analysis.

After each LEP recording, all subjects were asked to rate laser pain by using a 101-point visual analog scale (VAS), in which “0” corresponded to no pain and “100” to the worst pain one may conceive.

### 2.3. LEP Analysis

Scalp LEPs include two main components: (1) the N1 potential, which is recorded in the temporal region contralateral to the stimulated side, and (2) the N2-P2 complex, which shows its maximal amplitude on the vertex (Cz). For all LEP components, peak latencies were measured. The peak-to-peak N2-P2 amplitude was measured. Because in labeling the N1 reponse a certain difficulty may be caused by noise, the N1 amplitude was calculated offline by referring the temporal electrode contralateral to the stimulated side (T3 or T4) to the Fz lead [14].

### 2.4. Statistical Analysis

The following variables were included in the statistical analysis: VAS score, latencies, and amplitudes of N1, N2, and P2 potentials. All measures were compared between groups and within groups. Comparison between groups (patients vs. controls) was performed for each site of stimulation (hands and feet) and each stimulation session (day and night). Moreover, within each group (patients and controls), we compared the results of daytime vs. nighttime trials for each site of stimulation (hands and feet). Lastly, in order to evaluate modifications of the latencies and amplitudes of all LEP components between the sessions and compare these modifications between patients and controls, all neurophysiologic measures obtained in nighttime session were expressed as percentages of the corresponding values recorded in daytime session, according to the formula:

(1) $$\begin{array}{c} \text{night}‐\text{to}‐\text{day variation}=\frac{\text{night result}}{\text{day result}}\times 100. \end{array}$$

The statistical analysis was performed in successive steps. First, the normality of the distribution of all variables by means of the Shapiro–Wilk's test was verified; then, nonparametric tests (Mann–Whitney *U*-test) were applied to comparisons between nonnormal distributed parameters; lastly, the parametric Student's *t*-test, or the one-way analysis of variance (ANOVA) was used to compare normal distributed variables.

The significance level was set at *p* < 0.05. In case of multiples comparison, in order to avoid family-wise type-I errors, a formal Bonferroni correction was applied to each family of comparisons, by dividing the limit of significance by the number of comparisons (for VAS score, N1 and N2-P2 latencies, and amplitudes, four comparisons were made: (1) controls during day vs. controls during night, (2) patients during day vs. patients during night, (3) controls vs. patients during day, and (4) controls vs. patients during night; therefore, the significance level was set at *p*=0.05/4=0.0125). Statistics were performed using the SYSTAT 12 software, version 12.02.00 for Windows® (copyright SYSTAT® Software Inc. 2007).

## 3. Results

### 3.1. Psychophysical Data

The mean VAS pain rating scores obtained in day and night sessions in patients and controls are reported in Table 3. In the RLS group, compared to controls, we found a significant increase of VAS score after foot (patients: 55.64 ± 16.45; controls: 34.73 ± 15.74  *U*-test =21.0; *p* = 0.009) and hand stimulation (patients: 55.91 ±12.93; controls: 31.64 ± 19.76; *U*-test = 18.0; *p* = 0.005) during night session.

### 3.2. Neurophysiological Data

Nerve conduction study, somatosensory-evoked potentials, and motor-evoked potentials were normal in all RLS patients and controls. Polysomnographic data are consistent with a severe RLS in all patients; in particular, all patients showed high sleep-onset latency (Table 1).

**Figure 1:**
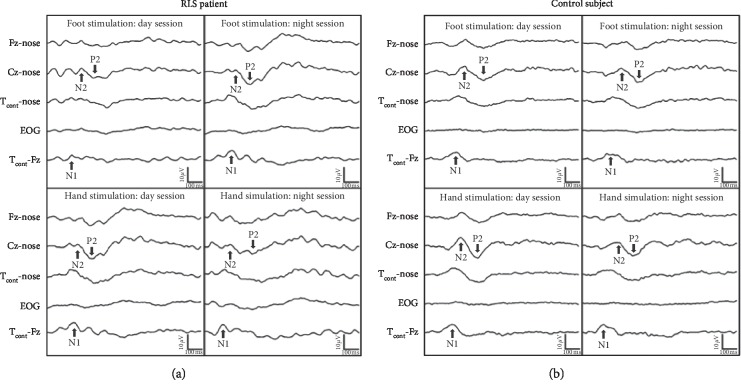
Laser-evoked potentials (LEP) obtained after foot and hand stimulation in an RLS patient (a) and a control subject (b). The figure shows, in RLS patient, that both A*δ*-N1 and A*δ*-N2-P2 amplitudes after foot stimulation are increased during night when compared to daytime recordings. Conversely, LEP amplitude after hand stimulation results in unmodified night and day stimulation. In control subject, LEP amplitudes are substantially unchanged in two sessions after foot and hand stimulation.

Reproducible N1 response and biphasic N2-P2 complex were recorded in all our patients and control subjects. *Within-groups* (day vs. night) and *between-groups* (controls vs. patients) comparisons of LEP latencies and amplitudes did not show significant differences (Table 4). Lastly, the night/day ratios of all LEP components were compared between patients and controls. The N1 and N2-P2 amplitude night/day ratios after foot stimulation were increased in patients (N1: patients: 133.91 ± 50.42%; controls: 83.74 ±34.45%; *U*-test = 82.0; *p*=0.016; A*δ*-N2-P2: patients: 119.15± 15.56%; controls: 88.42 ± 23.41%; *U*-test = 106; *p*=0.003) (Figure 1) (Table 5).

## 4. Discussion

The A*δ*-fiber responses recorded after foot and hand stimulation in all our patients did not differ from those obtained in control subjects. This result suggests that RLS patients do not have a small fiber neuropathy. However, while RLS patients showed increase of the LEP amplitudes and pain rating values to foot stimulation in the night compared to the day session, in control subjects, LEP amplitudes and VAS values decreased during the night. This suggests that RLS patients present circadian modifications in the pain system, which are not present in healthy controls. These circadian modifications involve both A*δ*-N1 and A*δ*-N2-P2 complex responses. Since these potentials have a different functional meaning, our findings support the involvement of both the sensory-discriminative [15] and affective-emotional components of pain [16].

### 4.1. Nociceptive System in RLS

Impairment of the nociceptive system in RLS has been hypothesized and investigated [17]. The only previous LEP study performed in RLS patients [12] reported normal responses to nociceptive fibers stimulation. However, RLS patients were examined only during the day; therefore, the circadian rhythm of the symptoms characterizing this disorder was not considered [12]. Psychophysiological studies have reported controversial results. In idiopathic RLS, Schattschneider et al. found normal temperature perception thresholds reflecting small fiber integrity [6]. Conversely, Stiasny-Kolster et al. [9] described pinprick hyperalgesia, reverted by l-DOPA treatment and associated with functional sensory loss [9]. Hyperalgesia, not associated with mechanical allodynia, was interpreted as an atypical form of central sensitization [9]. The authors interpreted the RLS symptoms as expression of the flexor reflex, a circuit that represents a key structure in the RLS phenomenology. Flexor reflex is organized as a protective device producing nociceptive withdrawal driven by A*δ* afferents. In this model, the interneuronal network mediating the withdrawal responses is connected with peripheral afferents and motor pathways. In this way, overlapping neural systems are “parsimoniously devoted to perform apparently unrelated actions such as nociceptive withdrawal and walking. Thus, this structure encompasses the essential substrate for the distinctive manifestations of RLS, both motor (urge to move) and sensory (dysesthesia) [18].

### 4.2. Circadian Modifications of Pain

Circadian oscillations in the pain system function are debated in healthy subjects. While a number of human studies on experimental pain documented significant circadian variation in pain perception, other studies failed in documenting any clinically relevant circadian variation in pain perception [19]. In a psychophysiological study, Strian et al. [20] could only detect small diurnal variations, characterized by high interindividual variability, which were “*not sufficient to explain the variations seen in clinical pain*.” In a more recent study with repeated sessions of LEP recordings in normal subjects, LEPs latencies did not show significant modifications in nocturnal versus diurnal recordings [21]. Our findings in the control group confirm that healthy subjects present only minimal or absent circadian fluctuation of the pain systems, thus supporting the teleological sentence of Bachmann et al., according to whom “the role of pain as a physiological protection makes significant diurnal alterations in pain perception in healthy humans unlikely” [21].

On the other hand, circadian rhythms in the occurrence and intensity of pain may be present in various pathologic conditions, such as migraine, rheumatoid arthritis, toothache, cancer, and intractable pain [19]. Also, in fibromyalgia there is a clear diurnal rhythmicity in pain perception [22].

Our RLS patients showed higher LEP amplitude and higher pain perception to foot stimulation during the night than during the day session. These circadian amplitude modifications involved both the N1 and N2-P2 responses. Intracerebral LEP recordings demonstrated that the N1 potential is generated in the secondary somatosensory area (SII area) [23], while the N2-P2 complex is originated from the anterior cingulate cortex (ACC) [24]. In particular, the P2 potential may be considered as the main marker of the genuine ACC activity, while other neural sources, such as the bilateral SII area [25], insula [15, 26] and, maybe, primary somatosensory area (SI area) [27], contribute to the N2 response generation. As compared to the P2 potential, the N1 response is less reduced in amplitude by distraction from the laser stimulus; thus, it has been linked to the sensory-discriminative component of pain [15]. On the contrary, the vertex LEP components, in particular the P2 potential, are extremely sensitive to cognitive factors, e.g., distraction from the painful stimulus, and are therefore thought to represent the neurophysiological counterpart of the attention-affective-emotional aspect of pain experience [16]. Given the fact that in our patients both the N1 and the N2-P2 amplitude underwent the same circadian modification, these results suggest that both sensory-discriminative [15] and attention-affective-emotional components of pain experience [16] undergo similar modifications. Since the N1 and the P2 potentials are generated by parallel spinal pathways [28], the spinal cord, where both pathways ascend closely, may be the site of this change. However, we cannot exclude the fact that other regions of the central nervous system may be involved.

### 4.3. Mechanisms of Circadian Fluctuation of Pain in RLS

In RLS, the circadian variations of the symptoms are probably related to the molecular mechanisms involved in its development. There are 3 main hypotheses to explain the sensory symptom fluctuations. First, dopaminergic systems play a major role in RLS. Dopaminergic projections to the spinal cord originate supraspinally in dorsoposterior hypothalamic area A11 [29]. The A11 axons primarily arrive to the dorsal horn, and project onward to the motoneuronal site [30]. The A11-diencephalic dopaminergic nucleus provides the main descending dopaminergic control of the spinal tract [31]. The A11 diencephalo-spinal pathway is known as a crucial structure for pain control at the spinal cord level [32]. Two elegant studies, in animal models, showed that A11 cell group in the hypothalamus is directly involved in descending control of pain [33, 34]. Clemens et al. suggested that in RLS, a dysfunction of the dopaminergic A11 neurons could shift the descending control to excitation with an increased sympathetic drive, leading to an aberrant activation of high-threshold muscle afferents [35]. Moreover, the A11-diencephalic dopaminergic nucleus has a close anatomical relationship with the suprachiasmatic nucleus, a key structure for circadian rhythms [36], and represents a relay between this and the spinal cord [3]. Therefore, the hypothesis can be made that in RLS, there is a fluctuating disinhibition of the nociceptive system, due to an impairment of the descending control structures. Second, a dysfunction in the production of melatonin, a hormone that has been associated with circadian modification of symptoms in other conditions, such as migraine and cluster headache [37], could have a role. Third, there could be a contribution of the cortical excitability variations. Circadian modifications of the cortical excitability have been described in normal subjects [38] and in RLS, both in humans [39] and in animal models [40]. In this view, the nocturnal increase in corticospinal excitability in RLS could account for both the motor disturbances and the increased pain sensitivity.

We have to mention a limitation of our study. Indeed, most our patients showed a high periodic limb movement (PLM) index. Therefore, we cannot be sure to what extent our findings could be related to PLM, rather than to RLS. However, PLM have been found in 80–89% of RLS patients [41]; thus, the dissociation between RLS and PLM is very difficult to investigate.

## 5. Conclusions

To the best of our knowledge, this is the first study evaluating nociceptive system in RLS patients, according to the circadian pattern of the symptoms. Our study confirms the structural integrity of A*δ* nociceptive system in idiopathic RLS. However, RLS patients show an abnormal circadian modification of A*δ*-fiber pathway excitability, possibly due to an impairment of the hypothalamic dopaminergic projections to the spinal cord.

## Figures and Tables

**Table 1:** Clinical and PSG data in RLS patients.

Patient	Sex	Age	Duration	IRLSSG score	Sleep latency (min)	PLM index (events/h)	Comorbidity
#1	M	22	4	36	61	79	HyperCKemia
#2	F	30	3	29	304	59	Overweight
#3	F	68	12	30	20	14	
#4	F	70	40	35	75	121	
#5	M	64	30	27	13	25	
#6	M	77	5	29	12	111	
#7	M	40	1	23	18	5	
#8	M	27	1	27	47	96	
#9	M	58	40	32	21	156	Glaucoma
#10	F	61	7	33	13	23	
#11	M	68	2	29	103	247	
*Mean*		53.2	13.2	30.0	62.3	85.1	
*SD*		19.6	15.6	3.8	85.7	72.9	

Polysomnographic data. Sleep latency: sleep-onset latency; PLM index: periodic limb movement index (number of events per hour).

**Table 2:** Control subjects.

CTR	Sex	Age	Sleep latency (min)	PLM index (events/h)
#1	M	60	17	5
#2	F	71	18	18
#3	M	49	11	13
#4	M	38	21	3
#5	M	74	9	0
#6	F	58	20	4
#7	F	64	25	4
#8	F	29	7	6
#9	M	71	19	4
#10	M	21	11	9
#11	M	74	14	16
*Mean*		55.4	15.6	7.5
*SD*		18.7	5.6	5.8

Polysomnographic data. Sleep latency: sleep-onset latency; PLM index: periodic limb movement index (number of events per hour).

**Table 3:** VAS pain rating score.

	Foot		Night vs. day	Hand		Night vs. day
	Night	Day		Night	Day	
RLS	55.64 ± 16.45	49.91 ± 12.77	*U* test: 68.5 *p*=0.599	55.91 ± 12.93	51.36 ± 10.92	*U* test: 72.5 *p*=0.430

CTR	34.73 ± 15.74	39.18 ± 19.49	*U* test: 50.5 *p*=0.511	31.64 ± 19.76	40.91 ± 20.31	*U* test: 54 *p*=0.669

RLS vs. CTR	*U* test: 21 *p*=0.009^*∗*^	*U* test: 38.5 *p*=0.148		*U* Test: 18 *p*=0.005^*∗*^	*U* test: 42.5 *p*=0.237	

**Table 4:** Neurophysiological data.

		Foot		Night vs day	Hand		Night vs day
		Night	Day		Night	Day	
N1 latency (ms)	RLS	149.5 ± 32.8	165.2 ± 34.2	*U* test: 37.5 *p*=0.218	151.7 ± 36.6	155.7 ± 31.8	*U* test: 54 *p*=0.669
	CTR	172.4 ± 30.0	174.1 ± 30.0	*U* test: 51 *p*=0.940	143.6 ± 24	152.3 ± 33.2	*U* test: 46 *p*=0.340

	RLS vs. CTR	*U* test: 29 *p*=0.112	*U* test: 45 *p*=0.481		*U* test: 67.5 *p*=0.646	*U* test: 71 *p*=0.490	

N1 amplitude (*μ*V)	RLS	3.1 ± 1.1	2.7 ± 2.4	*U* test: 73.5 *p*=0.193	3.8 ± 2.6	3.6 ± 2	*U* test: 54 *p*=0.670
	CTR	3.7 ± 1.5	4.8 ± 2.0	*U* test: 31.5 *p*=0.162	4.5 ± 2.7	5.2 ± 1.5	*U* test: 43 *p*=0.250

	RLS vs. CTR	*U* test: 41 *p*=0.496	*U* test: 21 *p*=0.017		*U* test: 48 *p*=0.412	*U* test: 34 *p*=0.082	

N2 latency (ms)	RLS	226.7 ± 46.8	238.9 ± 54.4	*U* test: 50 *p*=0.725	218.8 ± 54.4	234.2 ± 56.4	*U* test: 59 *p*=0.922
	CTR	236.8 ± 26.0	223.2 ± 33.1	*U* test: 62 *p*=0.621	201.8 ± 31.2	197.5 ± 36.5	*U* test: 67 *p*=0.669

	RLS vs. CTR	*U* test: 57 *p*=0.888	*U* test: 66 *p*=0.438		*U* test: 78 *p*=0.250	*U* test: 86 *p*=0.094	

P2 latency (ms)	RLS	396.6 ± 83.4	372.7 ± 60.9	*U* test: 79 *p*=0.224	351.8 ± 60.4	365.8 ± 72.6	*U* test: 84 *p*=0.123
	CTR	364.8 ± 29.9	353.2 ± 31.0	*U* Test: 71.5 *p*=0.470	318.8 ± 37.9	322.4 ± 41.7	*U* Test:56.5 *p*=0.793

	RLS vs. CTR	*U* test: 79 *p*=0.224	*U* test: 75.5 *p*=0.324		*U* test: 84 *p*=0.123	*U* test: 83 *p*=0.140	

N2-P2 amplitude (*μ*V)	RLS	13.6 ± 5.2	11.5 ± 4.0	*U* Test: 74.5 *p*=0.358	10.7 ± 5.1	11.1 ± 4.7	*U* test: 60 *p*=0.974
	CRL	12.4 ± 7.1	15 ± 9.0	*U* test: 49 *p*=0.450	14.1 ± 8.6	16.9 ± 11.1	*U* test: 52 *p*=0.577

	RLS vs. CTR	*U* test: 74 *p*=0.375	*U* test: 50 *p*=0.490		*U* test: 47 *p*=0.375	*U* test: 39 *p*=0.158	

**Table 5:** Neurophysiological data: night/day ratio.

		Foot		Hand	
N1 latency	RLS	94.2 ± 10.6	*U* test: 69 *p*=0.151	98.8 ± 21.7	*U* test: 68 *p*=0.622
	CTR	99.8 ± 12.5		95.7 ± 10.3	

N1 amplitude	RLS	133.9 ± 50.4	*U* test: 82 *p*=0.016^*∗*^	135.2 ± 124.5	*U* test: 65 *p*=0.768
	CTR	83.7 ± 34.5		87.5 ± 41.4	

N2 latency	RLS	100.3 ± 13.7	*U* test: 35 *p*=0.257	95.2 ± 16.3	*U* test: 52 *p*=0.577
	CTR	106.0 ± 12.0		103.5 ± 14.9	

P2 latency	RLS	106.2 ± 12.6	*U* test: 69 *p*=0.577	96.8 ± 6.6	*U* test: 52 *p*=0.577
	CTR	103.6 ± 8.2		99.4 ± 9.1	

N2-P2 amplitude	RLS	119.2 ± 15.6	*U* test: 106 *p*=0.003^*∗*^	100.2 ± 35.5	*U* test: 76 *p*=0.309
	CTR	88.4 ± 23.4		86.2 ± 22.6	

## Data Availability

The neurophysiological laser-evoked potential data used to support the findings of this study are available from the corresponding author upon request.
